# Assessing the Role of Compression Eye Patches for Orbital Surgery: A Comparative Analysis of 10 Patients Undergoing Bilateral Orbital Decompression Surgery

**DOI:** 10.7759/cureus.82113

**Published:** 2025-04-11

**Authors:** Ryo Kikuchi, Tomoyuki Kashima

**Affiliations:** 1 Ophthalmology, Oculofacial Clinic Group, Tokyo, JPN

**Keywords:** compression eye patches, orbital fat decompression, postoperative care, retrobulbar hemorrhage, vision loss

## Abstract

Introduction

Postoperative eye patches are commonly used in orbital surgery to prevent bruising and edema, but there are only a few studies on their usefulness. We have performed bilateral orbital fat decompression as a surgical treatment for thyroid eye disease, which is very useful for relieving orbital pressure and improving functional and cosmetic outcomes. In this study, we compared the postoperative outcomes of patients with and without a postoperative compression eye patch and evaluated its effect on postoperative status based on bilateral orbital fat decompression.

Methods

This retrospective study analyzed 10 patients (five with compression eye patches and five without) who underwent bilateral orbital fat decompression surgery at Oculofacial Clinic Tokyo. A transconjunctival approach was employed, with no sutures used for wound closure. Outcomes, including retrobulbar hemorrhage, bruising, wound healing, edema, and patient-reported comfort, were assessed through photo documentation and clinical follow-ups at 24 hours, one week, and one-month post-surgery.

Results

Both groups exhibited comparable outcomes with no significant differences in postoperative bruising, edema, or complications. No retrobulbar hemorrhage, infection, or wound dehiscence was observed in either group. Patients without compression eye patches reported higher comfort levels and greater satisfaction with their postoperative experience. Visual acuity and intraocular pressure remained stable, and all patients experienced reduced proptosis.

Conclusion

The findings suggest that compression eye patches may not be essential in the postoperative care of orbital fat decompression surgery. Their omission improves patient comfort without increasing complications, providing evidence to reconsider their routine use. Large prospective studies are required to confirm these findings and refine postoperative care protocols.

## Introduction

The application of compression eye patches is reported to minimize complications like retrobulbar and subcutaneous hemorrhage [1], but there are only a few reports on their usefulness for orbital surgery. Similarly, some studies advocate the use of cold packs for reducing postoperative edema [2, 3]. Orbital fat decompression surgery is a well-established procedure for treating thyroid eye disease [4], and it aims to relieve pressure in the eye socket by removing fat, thereby enhancing patient comfort and improving visual function. To evaluate the necessity and efficacy of employing compression eye patches for orbital surgery as a standard postoperative care measure, this study aims to examine whether the absence of compression eye patches compromises the postoperative outcomes, specifically focusing on the risks of retrobulbar and subcutaneous hemorrhage.

## Materials and methods

Study design and setting

This is a retrospective study conducted at Oculofacial Clinic Tokyo. All procedures followed were in accordance with the Declaration of Helsinki. The Institutional Review Boards of the Oculofacial Clinic Group approved the study protocol, and the study was registered with the Ethical Committee of the Oculofacial Clinic Group (Approval Number: 2023082701).

Participants

This study included 10 patients who underwent bilateral orbital fat decompression surgery between June and August 2023. The inclusion criteria were patients who underwent bilateral orbital fat decompression under general anesthesia simultaneously. Exclusion criteria were patients with a history of orbital or lower eyelid surgery and patients who had undergone other concomitant surgeries. The participants were divided into two groups based on postoperative care protocols:

Group 1 (with compression eye patches): This group consisted of five female patients with a mean age of 45.8 years (range: 32-66, SD: 13.9). All patients were diagnosed with thyroid eye disease.

Group 2 (without compression eye patches): This group included three female and two male patients with a mean age of 48.0 years (range: 29-65, SD: 13.6). Four patients were diagnosed with thyroid eye disease, and one had congenital proptosis.

None of the patients in either group were on oral antithrombotic medications.

Surgical procedure

The surgery was performed by several experienced ophthalmic surgeons affiliated with our institution, each using a transconjunctival approach. A lower eyelid conjunctival incision was utilized to access the orbital fat. Thorpe forceps were specifically used to isolate and remove the excess fat. Both superficial and deep-seated orbital fat were targeted, but the emphasis was mainly on the removal of deep-seated fat. No sutures were applied; the surgical sites were meticulously managed to allow for sutureless closure. Any cases were performed under a precision microscope.

Postsurgical care

After completing the surgery under general anesthesia, all patients were instructed to rest in bed before being discharged home.

Group 1 (with compression eye patches): The compression eye patches were applied immediately after surgery for two hours and were removed when the patient was discharged.

Group 2 (without compression eye patches): No compression eye patches were applied.

For both groups, patients were instructed to continue bed rest as much as possible and apply cold packs to minimize swelling until the next morning. An antibiotic ointment was applied to the conjunctival wound immediately after surgery and from the next day. Follow-up assessments were conducted the day after surgery, one week later, and one month later to monitor recovery, wound healing, and any potential complications.

Data collection

Medical records were reviewed to extract relevant data, including demographic information, surgical details, and postoperative outcomes. Specifically, we sought the occurrence of retrobulbar and subcutaneous hemorrhage, rate of wound healing, levels of postoperative edema, and self-reported comfort levels during the postoperative period.

Outcome measures

The primary outcome measures included the occurrence of retrobulbar and wound hemorrhage within the first 24 hours postoperatively. Secondary outcome measures included evaluating general healing status assessed during the one-month follow-up visit, assessing levels of postoperative edema, and considering patient comfort.

## Results

The details of the 10 patients who participated in this study are shown in Table 1. The volume of fat removed differed slightly between the two groups. In Group 1, which consisted of patients with compression eye patches, the amount of fat removed per orbit ranged from 2.0 to 4.2 ml, while in Group 2, which consisted of patients without compression eye patches, the range was 2.5 to 6.0 ml. There were no significant changes in visual acuity and intraocular pressure pre- and postoperatively. There was also a decrease in ocular proptosis in all patients (Table 2). All patients displayed satisfactory wound healing without any signs of infection or complications. The surgical sites appeared well-closed, and there were no instances of wound dehiscence or delayed healing.

**Table 1:** Demographic information of patients

Patient No.	Age	Sex	Disease	Comorbidity
Group 1				
1	37	Female	Thyroid eye disease	Graves' disease
2	32	Female	Thyroid eye disease	Graves' disease
3	48	Female	Thyroid eye disease	Graves' disease
4	46	Female	Thyroid eye disease	Graves' disease
5	66	Female	Thyroid eye disease	Graves' disease, Diabetes, Left eye diabetic macular edema
Group 2				
1	29	Male	Thyroid eye disease	Graves' disease
2	42	Female	Thyroid eye disease	Graves' disease
3	41	Male	Congenital proptosis	Nil
4	63	Female	Thyroid eye disease	Graves' disease, Glaucoma, Meniere's disease
5	65	Female	Thyroid eye disease	Graves' disease, Hypertension, Breast cancer

**Table 2:** Amount of fat removed, visual acuity (VA), intraocular pressure (IOP), and ocular proptosis (OP) before and after surgery OD = Oculus Dexter, OS = Oculus Sinister

Patient No.	Amount of fat removed (ml)		VA before surgery (log MAR)		VA after surgery (log MAR)		IOP before surgery (mmHg)			IOP after surgery (mmHg)		OP before surgery (mm)		OP after surgery (mm)	
	OD	OS	OD	OS	OD	OS	OD		OS	OD	OS	OD	OS	OD	OS
Group 1															
1	2.7	2.5	-0.10	-0.10	19	18	-0.10	-0.10		15	16	21	21	20	20
2	3.2	3.1	-0.10	-0.10	18	18	-0.10	-0.10		21	17	18	18	19	20
3	2.0	3.0	-0.10	-0.10	19	17	-0.10	0		17	18	15	17	13	14
4	4.0	3.9	-0.10	-0.10	20	19	0	0		17	16	22	22	17	17
5	4.2	4.2	-0.10	1.0	17	22	0	0.80		20	20	21	20	14	15
Group 2															
1	6.0	5.0	-0.10	0	14	13	-0.10	0		14	14	20	20	15	15
2	3.2	2.5	-0.10	-0.10	15	17	-0.10	-0.10		14	18	20	20	17	17
3	4.4	4.4	-0.10	-0.10	22	22	-0.10	-0.10		18	16	18	18	15	15
4	4.0	4.5	1.0	0.20	7.0	11	1.0	0.20		8.0	9.0	23	22	20	21
5	3.2	3.2	-0.10	-0.10	16	16	-0.1	-0.10		18	19	20	20	18	18

None of the 20 orbits exhibited signs of retrobulbar or subcutaneous hematoma within the first 24 hours postoperatively. Postoperative edema was minimal among all participants, indicating very low levels of swelling during the postoperative period. Comparison of facial photographs of both groups on the day after surgery showed no difference in bruising (Figures 1-10).

**Figure 1:**
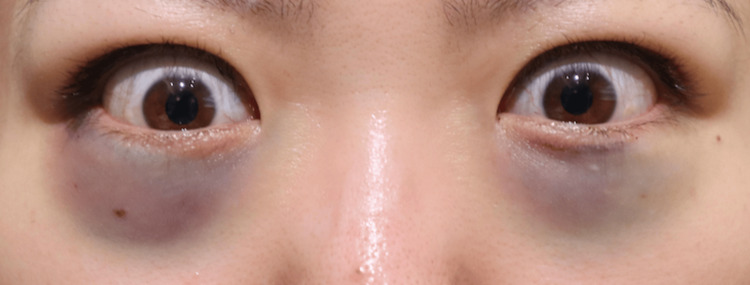
Group 1 Patient No. 1

**Figure 2:**
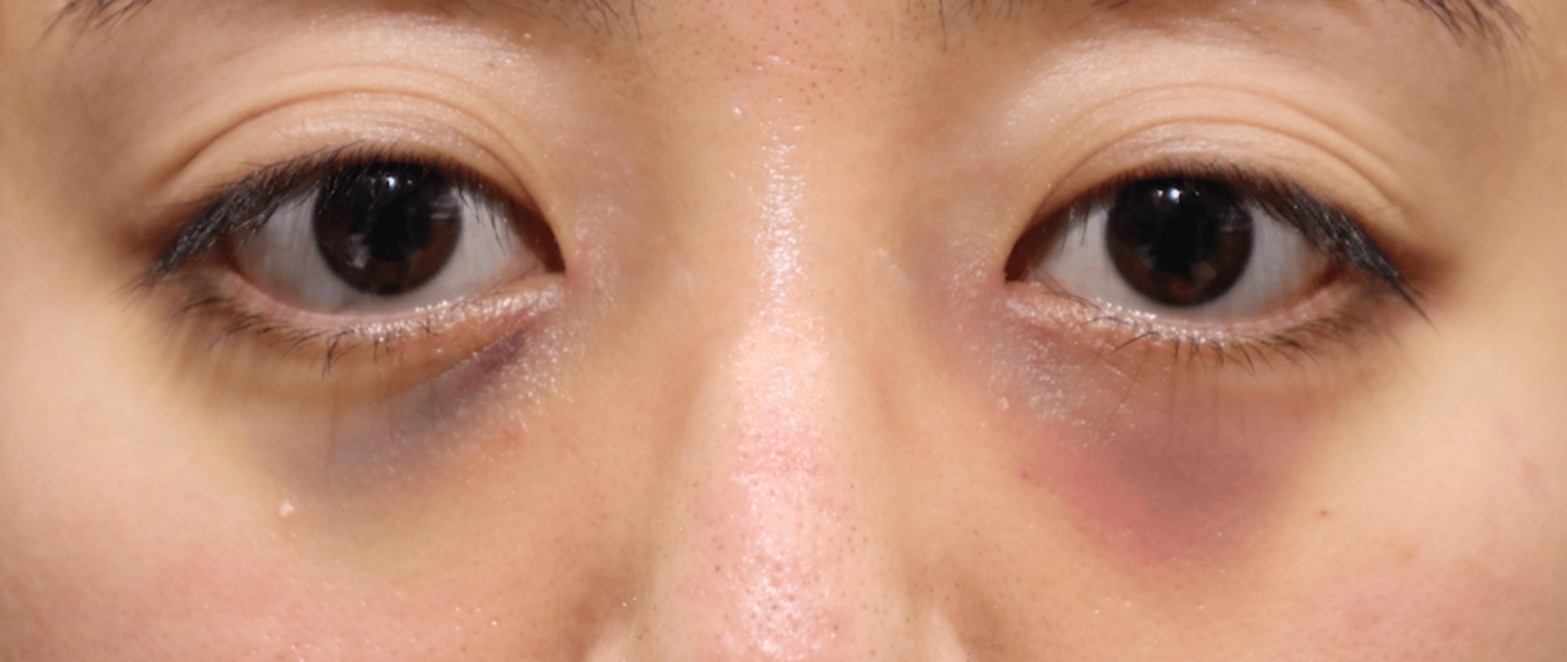
Group 1 Patient No. 2

**Figure 3:**
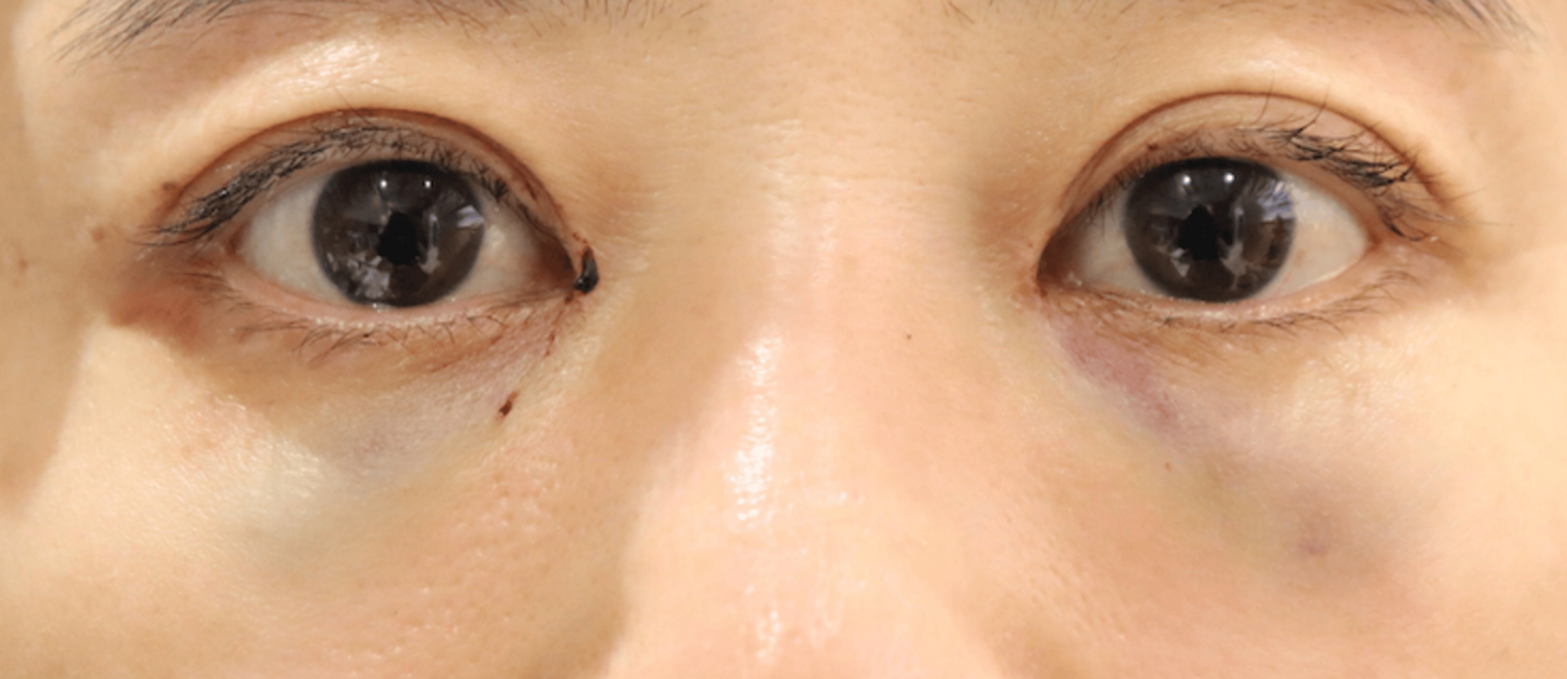
Group 1 Patient No. 3

**Figure 4:**
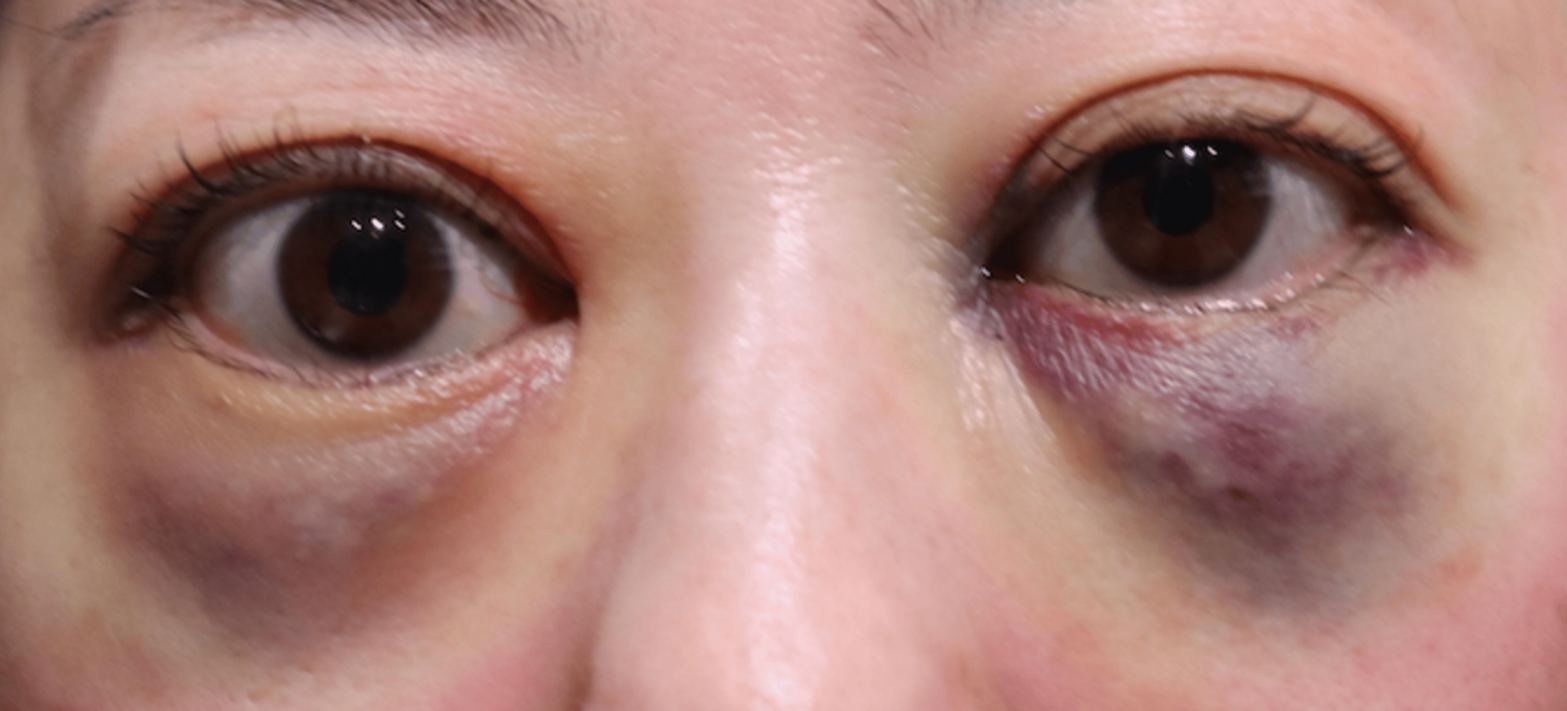
Group 1 Patient No. 4

**Figure 5:**
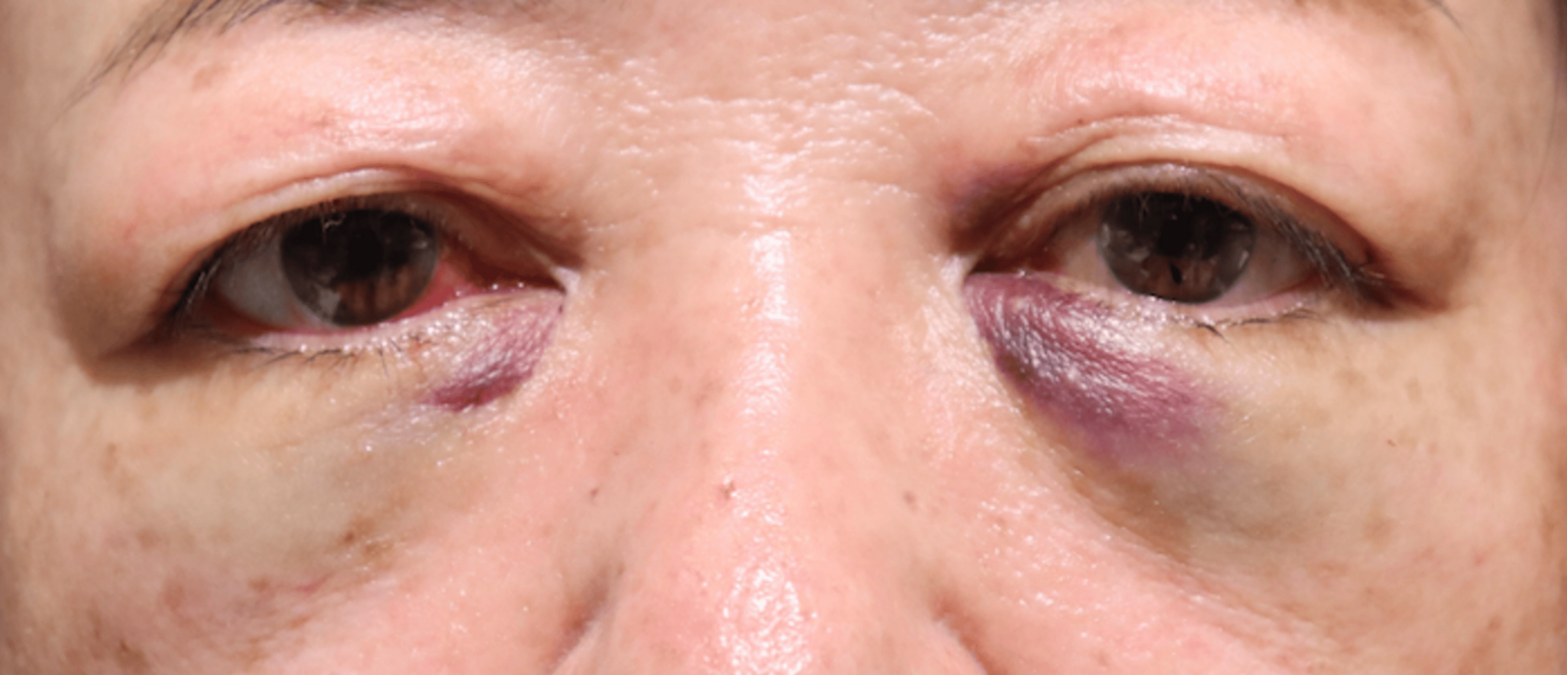
Group 1 Patient No. 5

**Figure 6:**
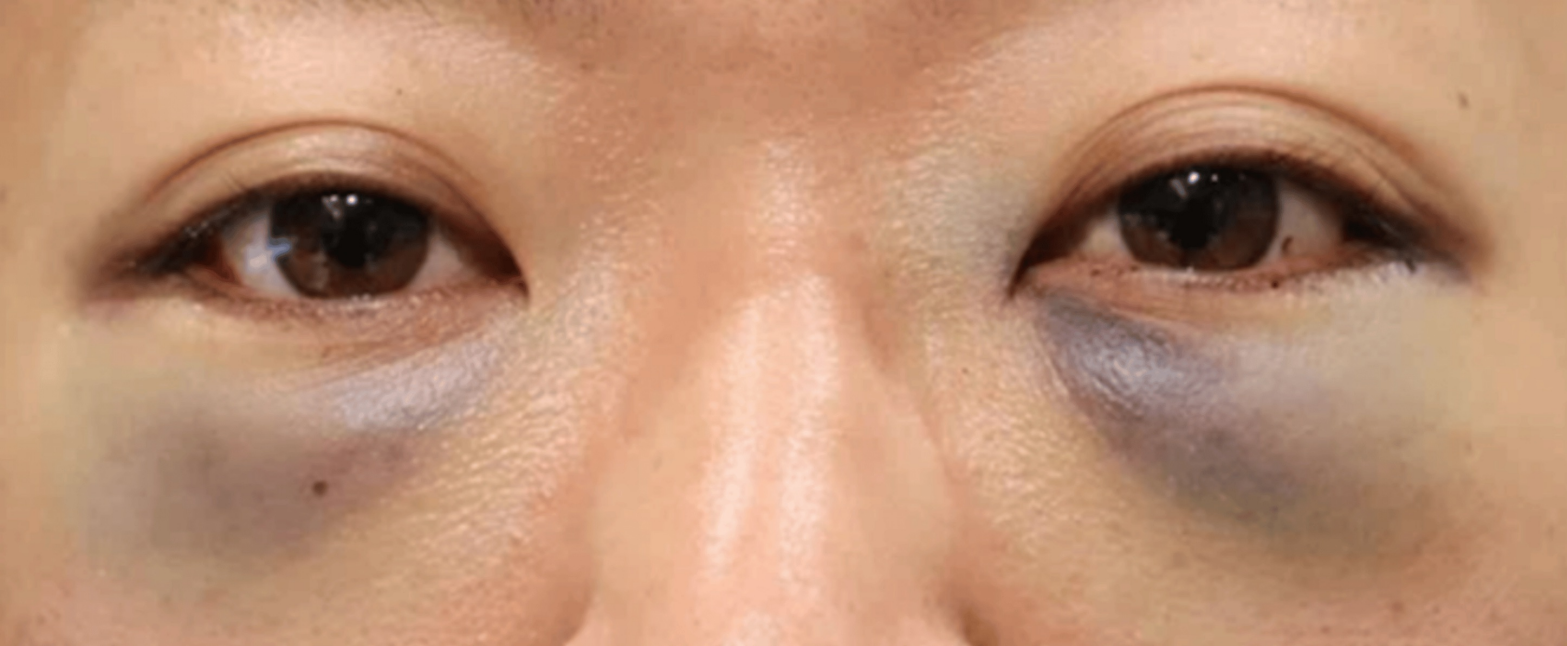
Group 2 Patient No. 1

**Figure 7:**
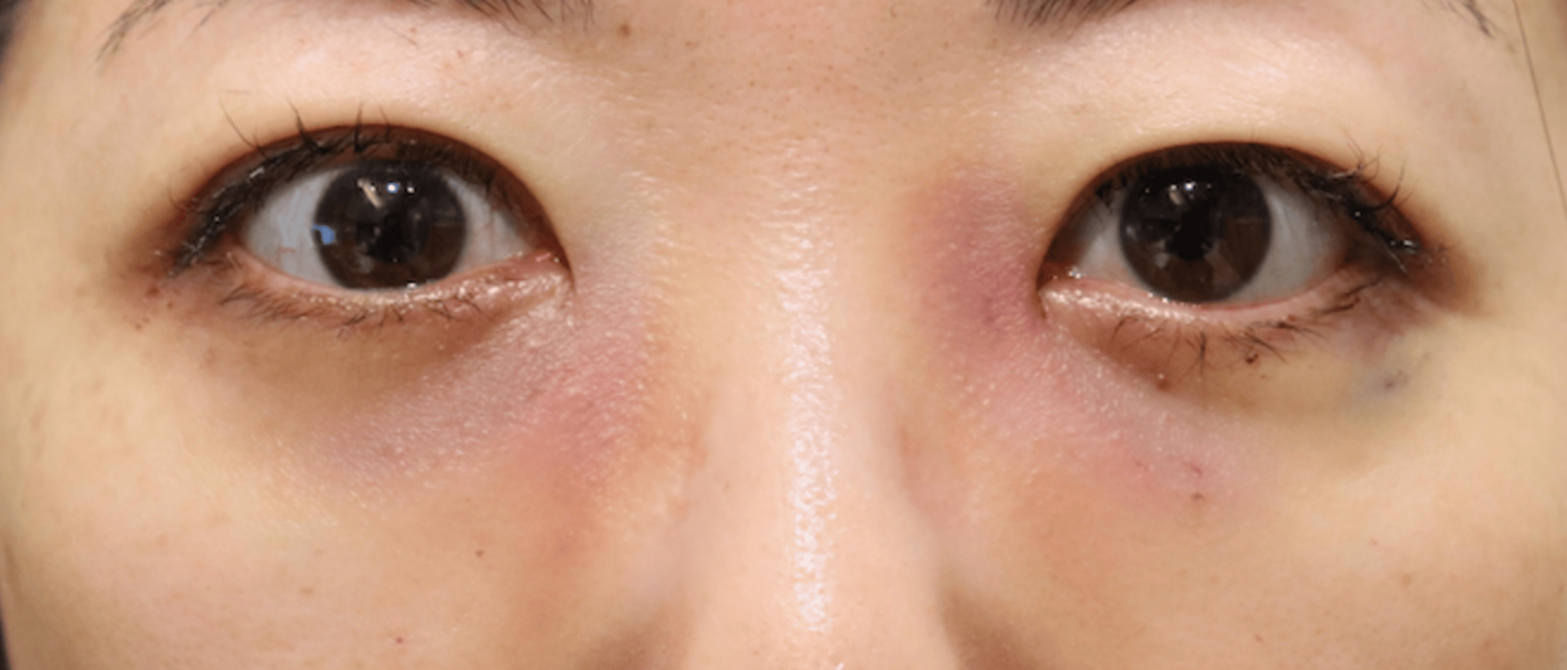
Group 2 Patient No. 2

**Figure 8:**
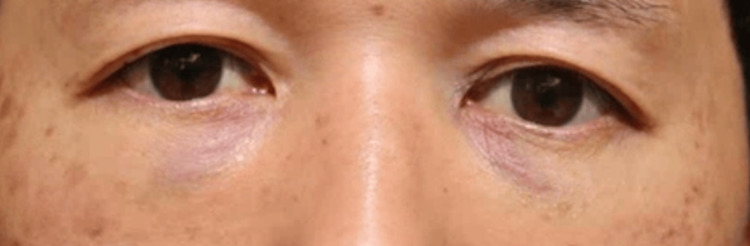
Group 2 Patient No. 3

**Figure 9:**
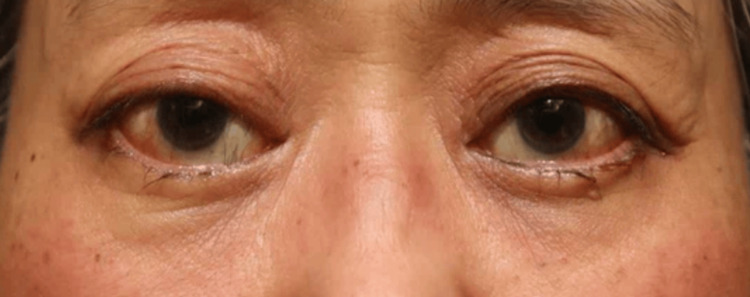
Group 2 Patient No. 4

**Figure 10:**
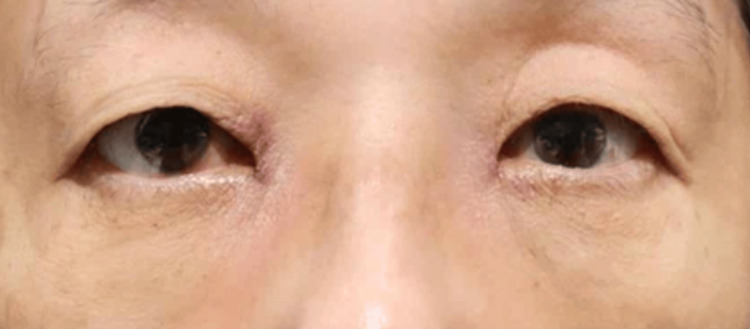
Group 2 Patient No. 5

Patient comfort

Self-reported comfort levels were notably high. When questioned during their one-month follow-up visits, all patients reported a high degree of comfort and were satisfied with their postoperative experience.

## Discussion

No previous reports clearly describe the incidence of postoperative retrobulbar hemorrhage after orbital decompression surgery. However, there are reports of retrobulbar hemorrhage in 1.2-3.2% of patients after orbital fracture surgery [5, 6], and blindness after orbital decompression has been reported to be 0-0.84% [7-12]. The results of our study revealed no significant differences in complications or surgical outcomes between the two groups, even in the absence of compression eye patches. No cases of retrobulbar hemorrhage, vision loss, or other major postoperative complications were observed in either group.

Preventive strategies, such as meticulous surgical techniques and intraoperative monitoring, have been proposed to mitigate the risks of visual impairment. Dohlman and Yoon advocate for standardized protocols that include intraoperative measures to minimize trauma and postoperative measures for early detection of complications [13]. Furthermore, Kansakar and Sundar emphasize the importance of immediate intervention in cases of retrobulbar hemorrhage or increased orbital pressure to prevent permanent vision loss [9].

The management of postoperative complications, particularly those involving optic nerve ischemia or compression, remains critical. Recent studies suggest that timely interventions, such as decompressive procedures or corticosteroid administration, can significantly improve outcomes [14, 15]. While these approaches were not necessary in our patient cohort, their application in high-risk cases could further enhance patient safety.

The role of a pressure eye patch is to increase the pressure within the tissue, thereby suppressing bleeding from blood vessels, assisting in hemostasis, and reducing postoperative swelling and inflammation. If the difference between the intravascular pressure and the tissue pressure is large, bleeding will continue until primary and secondary hemostasis - mediated by platelets and fibrin - occurs. As blood accumulates in the tissue, the tissue pressure rises, narrowing the gap with intravascular pressure, which reduces bleeding and eventually leads to hemostasis. By artificially elevating the tissue pressure, a pressure eye patch promotes early hemostasis and helps reduce postoperative internal bleeding and swelling. However, unlike wounds on the body’s surface, the orbital structure is shaped like a partially recessed, quadrangular, pyramid-like pocket. Because of this, even if a pressure eye patch is applied, the pressure may disperse and fail to achieve the desired compressive effect. Additionally, we must consider the disadvantages of compression eye patches, which prevent patients from immediately noticing changes in their visual acuity and visual field after surgery.

The absence of previous research on compression eye patch necessity can be attributed to several factors. Orbital diseases and surgeries are typically unilateral, which limits the feasibility of studying the necessity of compression eye patches in such cases. This is due to the difficulty in establishing control groups for comparison, as the unaffected side of each patient cannot provide equivalent baseline data. However, the bilateral orbital fat decompression surgeries performed at our institution provide a unique opportunity to directly compare outcomes between patched and unpatched eyes. This methodology minimizes inter-patient variability and allows for a more accurate assessment of postoperative outcomes.

Our case series provides preliminary evidence challenging the traditional postoperative use of compression eye patches in orbital fat decompression surgery. The absence of retrobulbar and subcutaneous hemorrhage in our sample suggests these patches may be unnecessary. These findings necessitate larger studies for validation but offer compelling avenues for further research and clinical practice changes.

The primary limitations of this study are its small sample size and the retrospective nature of the analysis. Although it included a comparative group, the small number of participants limits the generalizability of the findings. Large-scale prospective studies are needed to validate these results and further evaluate the role of compression eye patches in postoperative care for orbital fat decompression surgery.

## Conclusions

This study demonstrated that the omission of compression eye patches in postoperative care for orbital fat decompression surgery did not lead to adverse outcomes, such as retrobulbar hemorrhage or significant bruising. Both groups, with and without compression eye patches, achieved satisfactory recovery, suggesting that the use of these patches may not be essential. Moreover, avoiding compression eye patches improved patient comfort and facilitated earlier detection of potential complications. These findings challenge the traditional postoperative care protocols and highlight the potential for more patient-centered approaches.
